# Expression of Membrane-Bound CC Chemokine Ligand 20 on Follicular T Helper Cells in T–B-Cell Conjugates

**DOI:** 10.3389/fimmu.2017.01871

**Published:** 2017-12-21

**Authors:** Adrian Y. S. Lee, Dorothea Reimer, Annette Zehrer, Ming Lu, Dirk Mielenz, Heinrich Körner

**Affiliations:** ^1^Menzies Institute for Medical Research, University of Tasmania, Hobart, TAS, Australia; ^2^Department of Internal Medicine, Western Hospital, Footscray, VIC, Australia; ^3^Department of Medicine and Radiology, The University of Melbourne, Melbourne, VIC, Australia; ^4^Division of Molecular Immunology, Internal Medicine III, University Hospital Erlangen, Nikolaus-Fiebiger-Center, University of Erlangen-Nuremberg, Erlangen, Germany; ^5^Institute of Clinical Pharmacology, Anhui Medical University, Key Laboratory of Anti-Inflammatory and Immunopharmacology, Ministry of Education, Engineering Technology Research Center of Anti-inflammatory and Immunodrugs in Anhui Province, Hefei, China

**Keywords:** T–B cell communication, CC chemokine ligand 20, CC chemokine receptor 6, humoral immune response, T cells

## Abstract

The CC chemokine receptor 6 (CCR6) and its sole chemokine ligand CC chemokine ligand 20 (CCL20) display an emerging role in the coordination of humoral immune responses. Recent studies demonstrate a role of this chemokine axis in the migration of B cells to key immunological sites during an immune response, and facilitating the generation of high-quality antibodies. Very little, however, is known about CCL20 and its role in these functions. We undertook a preliminary investigation into the expression and function of CCL20 and demonstrate its well-noted upregulation in the spleen during immunization. Furthermore, we show that most follicular T helper (Tfh) cells can be CCR6^+^ and can produce CCL20. Surprisingly, CCL20 cannot only be found in the cytoplasm but also on the surface of these cells and their precursors. Analysis of T–B-cell conjugates revealed that mature Tfh cells, but not their precursors, are highly enriched in the conjugates. Further functional studies are needed to unravel the precise role of CCL20 in coordinating T and B cell interactions during the humoral immune response.

## Introduction

CC chemokine ligand 20 (CCL20) is emerging as an important mediator in the molecular orchestration of immunological homeostasis as well as the inflammatory and the humoral immune response (1, 2). CCL20 is expressed in endothelial cells (3), macrophages (4), and Th17 cells (5) and is upregulated during inflammation (6). The only known receptor for CCL20 is the CC chemokine receptor 6 (CCR6) which is found predominantly on dendritic cells (DC) (7), and on various lympocyte populations such as B cells, regulatory T cells (Tregs), Th17, and memory T cells (2). Mice genetically deficient for CCR6 demonstrate structural and functional deficits in mucosal immunity (8) highlighting the importance of this chemokine axis in homeostasis. Under inflammatory conditions, the regulation of CCR6 on DC, CD4^+^ T cell subpopulations, and B cells controls the migration of immune cells for correct positioning facilitating an efficient response (9, 10).

The cellular expression of CCL20 and its putative function in the humoral immune response are still not understood. In comparison, its partner CCR6 on B cells has been characterized and its dynamic expression has been found to orchestrate B cells and the germinal center (GC) response. B cells that leave the bone marrow to mature upregulate CCR6, and become highly responsive to CCL20—the precise reason of which has not been fully elucidated (9, 11). In CCR6-deficient mice, the GC response was quantitatively enhanced but at the expense of efficient and specific humoral immune responses (11, 12) and memory B cell responses (10). This suggests an important role for the CCR6–CCL20 chemokine axis in humoral immunity. To further investigate these findings, we decided to investigate the potential role for CCL20 in coordinating T and B cell interactions.

## Materials and Methods

### Mice and Immunizations

C57BL/6 WT and CCR6^−/−^ mice on a C57BL/6 background (B6.CCR6^−/−^) were sourced and generated as described (13). Animal experiments were approved by the University of Tasmania Animal Ethics Committee. Immunization was carried out by injecting 1.0 × 10^8^ sheep red blood cells (SRBC) suspended in sterile PBS intraperitoneally.

### Polymerase Chain Reaction (PCR)

Spleens from sacrificed mice were removed and homogenized in liquid nitrogen. Spleens were then added to 1 mL of TriReagent (Sigma-Aldrich, NSW, Australia) and RNA extraction carried out as per manufacturer’s instructions. cDNA was synthesized from total RNA using Tetro cDNA synthesis kit (Bioline, Alexandria, NSW, Australia). Template cDNA was stored at −20°C until further use. Quantitative real-time PCR was performed on cDNA templates using the SensiFAST SYBR No-Rox kit (Bioline). Results were normalized to β-actin (sense 5′- AGA GGG AAA TCG TGC GTG AC -3′ and anti-sense 5′- CAA TAG TGA TGA CCT GGC CGT -3′). The following primers were used: *Ccr6* (sense 5′- TGT CCT CAC CCT ACC GTT CTG -3′ and anti-sense 5′- TAC AGG CCA GGA GCA GCA T -3′), and *Bcl6* (sense 5′- CTG CAG ATG GAG CAT -3′ and anti-sense 5′- CGG CTG TTC AGG AAC -3′).

### Antibodies

The following rat anti-mouse antibodies and conjugations were obtained from BioLegend (Australian Biosearch, WA, Australia), BD Biosciences (Sydney, NSW, Australia), or eBioscience (Sydney, NSW, Australia) and used for flow cytometry: B220-Biotin (clone RA3-6B2), CD19-APC Fire 750 (6D5), CCR6-PE (29-2L17), CCR6-AF647 (140706), CD11b-PerCP-Cy5.5 (M1/70), CD11b-BV510 (M1/70), CD4-APC (RM4-5), CD4-PerCP Cy5.5 (RM4-5), CD8α-PB (53-6.7), CXCR5-Biotin (2G8), CXCR5-PerCP-Cy5.5 (2G8), PD-1-PE (J43), PD-1-PE-Cy7 (J43), TCR-αβ-PB (HM3628, Thermo Fisher Scientific Australia, Soresby, VIC, Australia), hamster IgG_1_-λ isotype-PE (G235–2356), and rat IgG_1_-κ isotype-FITC (eBRG1). Cy5-conjugated streptavidin (Jackson Immuno Research, Pennsylvania, PA, USA) was used as secondary reagent. Unlabeled CCL20 (114906) was obtained from R&D Systems (Sydney, NSW, Australia) and labeled with DyLight 488 Microscale Antibody Labeling Kit (Thermo Fisher Scientific, Australia) according to the manufacturer’s instructions.

### Flow Cytometry

Murine spleens were dissected and pushed through a 40 µm nylon cell strainer to obtain a single cell suspension. After washing, the cells were resuspended in 10 mL of red blood cell lysis buffer and left to incubate at room temperature for 10 min. For cells undergoing intracellular cytokine staining, 0.5 µL of 200 µg/mL PMA (Sigma-Aldrich) and 0.5 µL of 10 mM ionomycin (Thermo Fisher Scientific, Australia) were added to a 5 mL resuspension of the cells in RPMI medium (Thermo Fisher Scientific, Australia) and were incubated at 37°C for 1 h, 5% CO_2_. Following this, 1 µL of Golgi stop (BD Biosciences) (equivalent to 3.75 mM monensin) was added and the suspension incubated for further 3 h at 37°C.

Multicolor flow cytometry was performed on splenocytes using CyAn ADP and Gallios flow cytometers (Beckman Coulter, Inc., NSW, Australia). Post-acquisition analysis was performed using FlowJo v7.6 software (TreeStar, Inc., Ashland, OR, USA) and Kaluza 1.5a software (Beckman Coulter, Inc.). Fluorescent-activated cell sorting (FACS) was performed using the MoFlo Astrios cell sorter (Beckman Coulter, Inc.) with a sorting accuracy >95%.

For surface-bound chemokine studies, cells were blocked with Fc-receptor block (BD Biosciences) for 30 min on ice and antibody staining performed under sodium azide-free conditions. Fluorescent-minus-one and isotype controls were used to minimize non-specific binding and autofluorescence. To dissociate CCL20 from the surface cells were washed with acid as described (14). Briefly, cells were pelleted and resuspended in 200 µL ice-cold acid buffer for 2 min. Samples were neutralized with 5× volumes of R10 medium and cells were washed twice with FACS buffer (2% FCS, 0,01% NaN_3_ in PBS). In the following experiments, Fc-block and surface staining were performed as indicated.

### Statistics

Statistical analyses using the non-parametric two-tailed Mann–Whitney *U* test were performed and graphs constructed using Prism software v5 (GraphPad Software). Graph columns represent median with error bars representing range. The alpha value was set at 0.05. In our figures these have been summarized as: *p < 0.05, **p < 0.01, ***p < 0.001.

## Results and Discussion

### CCL20 Expression in Different Splenic Immune Cell Types

CC chemokine ligand 20 is an inflammatory chemokine that is upregulated by the pro-inflammatory cytokine TNF (3) or induced by TGF-β and IL-6, or IL-21 during T_H_17 cell differentiation (5). To examine whether CCL20 is involved in humoral immune responses, we immunized mice with SRBC, known to trigger strong GC responses. Splenocytes from unchallenged mice expressed virtually no CCL20 (Figure S1A in Supplementary Material). After immunization with SRBC splenocytes showed an upregulation of CCL20 mRNA in a time-dependent manner reaching approximately ninefold expression by day 7 (Figure S1A in Supplementary Material). Interestingly, *Ccl20* has a very low-constitutive expression in the spleen, relative to other organs such as Peyer’s patches (15) (Adrian Y. S. Lee and Heinrich Körner, unpublished data) suggesting a subordinate role in splenic immune homeostasis. In fact, the rapid and marked elevation of *Ccl20* post-immunization suggests it has a very specific role in the inflammatory response. Therefore, we examined the expression of CCL20 in this organ for subsequent experiments.

To identify possible sources for splenic CCL20, we sorted day 5 SRBC-immunized splenocytes and performed quantitative real-time PCR on major cellular subpopulations found in the spleen: B cells (B220^+^TCR-β¯), CD4^+^ T cells (B220¯TCR-β^+^CD4^+^), CD8^+^ T cells (B220¯TCR-β^+^CD8^+^), and miscellaneous CD11b^+^ cell populations (B220¯TCR-β¯CD11b^+^) which predominantly consists of NK cells, splenic macrophages, and granulocytes (4, 16). An earlier study (10) demonstrated very low-constitutive *Ccl20* expression in the CD4^+^ T cell compartment of the spleen and no expression in the CD8^+^ T cell or monocytic compartments. Our study, however, demonstrated *Ccl20* mRNA highly expressed in CD11b^+^ cells after activation (Figure S1B in Supplementary Material). In comparison, total splenic *Ccl20* expression was low suggesting a marked dilution by non-CCL20-producing cells, most likely the B cells. This is in line with early expression studies that found overall very low amounts of constitutive splenic *Ccl20* compared with other organs (15). However, we show a substantially upregulated expression induced upon immunization (Figure S1A in Supplementary Material) or challenge using bacterial lipopolysaccaride (data not shown).

### Intracellular CCL20 Expression in Different Subsets of CD4^+^ T Cells

**Figure 1:**
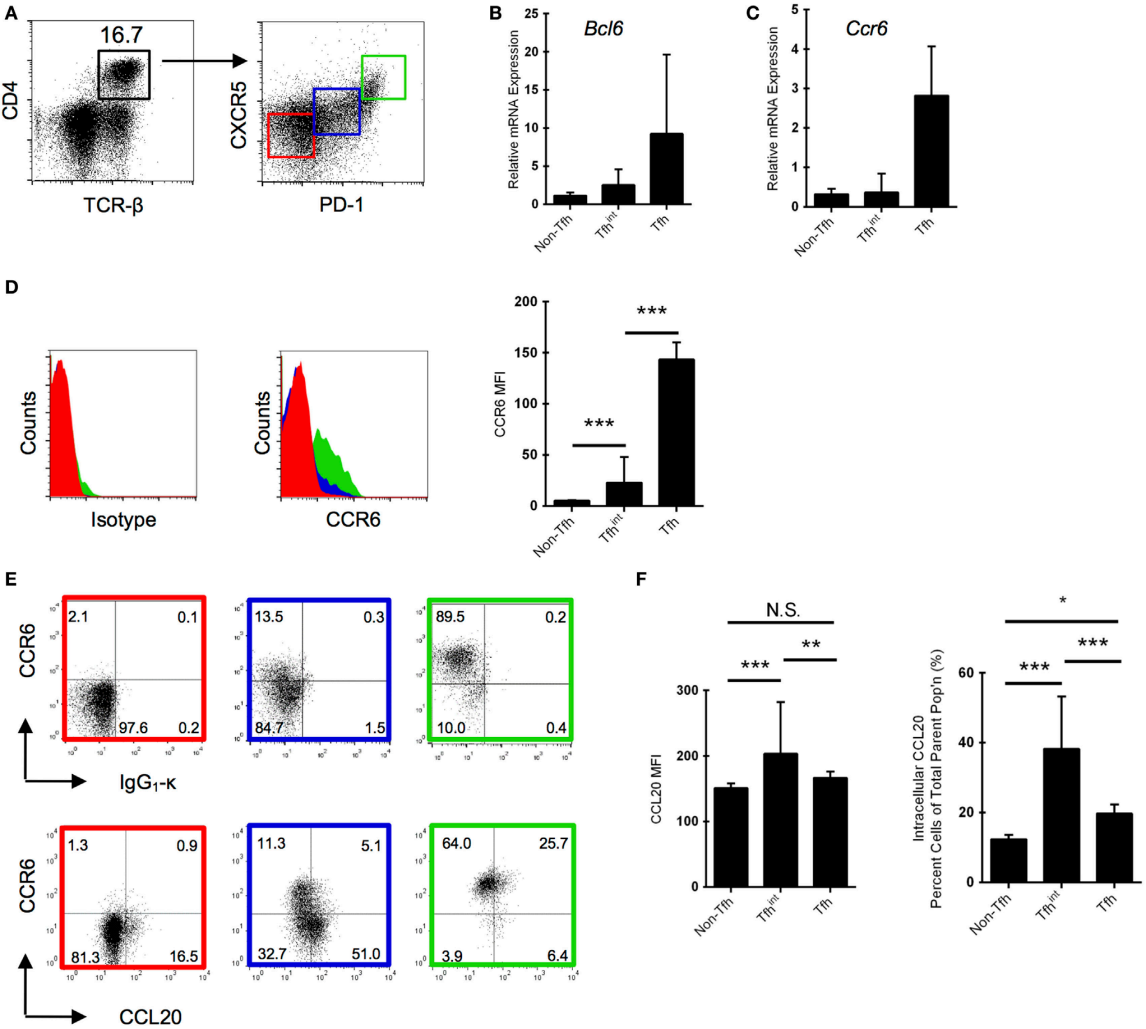
Follicular T helper (Tfh) cells express CC chemokine receptor 6 (CCR6) and are positive for CC chemokine ligand 20 (CCL20) protein. **(A)** Gating strategy for Tfh cells isolated from splenocytes by fluorescent-activated cell sorting. Non-Tfh cells (PD-1^−^CXCR5^−^) are marked in red, intermediary Tfh (Tfh^int^) (PD-1^lo^CXCR5^lo^) cells are marked in blue, and Tfh cells (PD-1^hi^CXCR5^hi^) marked in green. mRNA expression of *Bcl6* **(B)** and *Ccr6* **(C)** normalized to *Actb* in the three isolated Tfh populations. One representative experiment is shown. Results are representative of two experiments with five to six mice each (Bcl6 and Ccr6) with 5-day sheep red blood cells (SRBC)-immunized mice. Means between each column in **(B,C)** are not significant (*p* > 0.05). **(D)** CCR6 protein expression in the three T cell populations *via* flow cytometry using an isotype control (left panel). Mean fluorescence intensity (MFI) of CCR6 is also provided (right panel). **(E)** Differential CCR6 and intracellular CCL20/isotype protein expression in the three splenic T cell populations *via* flow cytometry in day 5 SRBC-immunized mice. CCL20 protein expression was quantified as MFIs and percentages **(F)**. CCL20 gating is based on an isotype control. Representative panels from five independent experiments with two to five mice each. *p < 0.05, **p < 0.01, ***p < 0.001.

CC chemokine receptor 6, the CCL20 receptor, modulates GC reactions and antibody affinity maturation, which appears to be regulated by follicular T helper (Tfh) cells (17). Thus, the CCR6/CCL20 axis might be involved in the GC response *via* Tfh cells (12) and we decided to focus our attention on CD4^+^ T cells, and specifically, Tfh cells. To initiate a strong humoral immune response, we used SRBC as a T cell-dependent antigen. We identified the Tfh population in spleen (Figure 1A) and sorted Tfh cells (defined as CD4^+^CXCR5^hi^PD-1^hi^) from SRBC-immunized mice (day 5 p.i.), an intermediate population Tfh^int^ (CD4^+^CXCR5^lo^PD-1^lo^) and the remaining CD4^+^ T cells (non-Tfh cells; CD4^+^CXCR5^−^PD-1^−^) (Figure 1A). These non-Tfh cells comprise other T helper subsets (e.g., Th1), Tregs, and memory T cells including memory Tfh cells (18). The classic effector Tfh cells were significantly increased following immunization, confirming their activation (Figure S2 in Supplementary Material). To validate the quality of the isolated cell populations, we subjected sorted Tfh, Tfh^int^, and non-Tfh cells to qRT-PCR for *Bcl6*, demonstrating a non-significant trend toward highest mRNA expression in the Tfh cells, moderate expression in the intermediary population, and lowest expression in the non-Tfh cells (Figure 1B). The fact that we detected more *Bcl6* in sorted CD4^+^TCRb^+^CXCR5^+^PD-1^+^ cells corroborates their identity as Tfh as shown previously (19). Similarly, we also show that mRNA expression of *Ccr6* tends to be highest in Tfh cells (Figure 1C), which corresponds to protein expression as determined by flow cytometry (Figure 1D).

The canonical CD4^+^CCR6^+^ T cell population has been attributed to Th17 cells (20, 21); however, we show approximately 90% of Tfh cells are positive for CCR6 (Figure 1E), consistent with our mRNA data for strong *Ccr6* expression in this population (Figure 1C). We performed intracellular cytokine staining and show that of the Tfh^int^ population, a marked proportion of CCR6^−^ cells are CCL20^+^, whereas very little CCR6^+^Tfh^int^ cells are positive (Figure 1E). In contrast, the CCL20 expression is less marked in Tfh cells, being present in approximately 30% of cells (Figure 1E). The dominant intracellular CCL20 expression in the intermediary population (Figure 1F) is congruent to microarray data that demonstrated that *Ccl20* is upregulated in intermediate population and down-regulated in Tfh cells (19). Interestingly, in a second study, CCL20 expression has also been described to be higher in Tfr cells than in regular Tfh cells (22).

### Surface Expression of CCL20 on Tfh Cells

**Figure 2:**
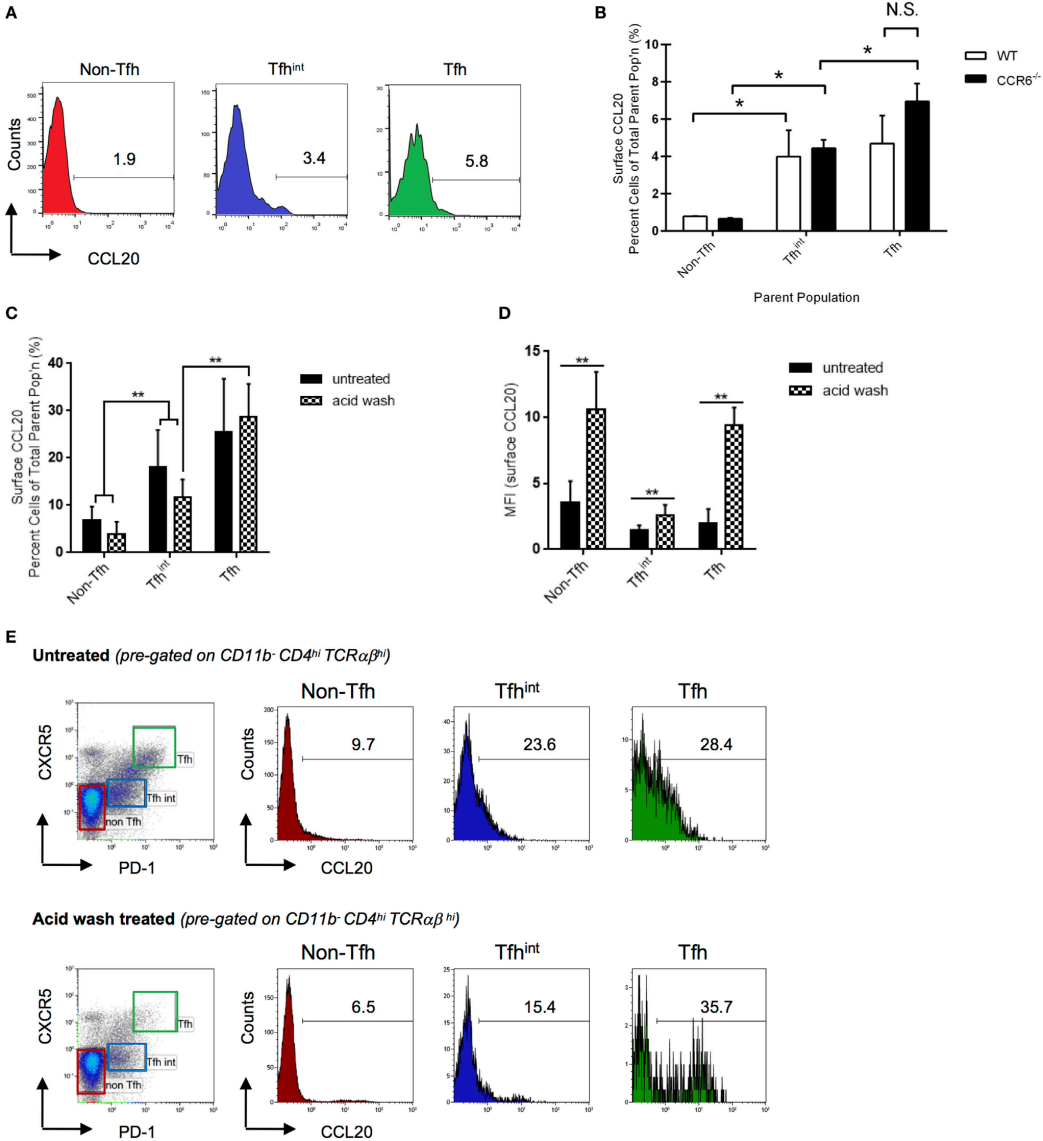
CC chemokine ligand 20 (CCL20) is found on the surface of splenic T cells. Surface-bound CCL20 was determined on T cells from sheep red blood cells-immunized WT and CCR6^−/−^ mice at day 5 after immunization. Non-follicular T helper (Tfh) cells (CD11b^−^CD4^hi^TCRαβ^hi^PD-1^−^CXCR5^−^), Tfh^int^ (CD11b^−^CD4^hi^TCRαβ^hi^PD-1^lo^CXCR5^lo^), and Tfh cells (CD11b^−^CD4^hi^TCRαβ^hi^PD-1^hi^CXCR5^hi^) were analyzed by flow cytometry. **(A)** Representative flow cytometry histograms of percent T cells with surface CCL20 in WT mice. The gate was made based on an isotype control. **(B)** Statistical analysis of the percentage of CCL20^+^ T cells in the total subpopulation comparing WT and CCR6^−/−^ mice. Representative bar graph from two experiments with 10 mice of each genotype in total. **(C)** Percent surface CCL20 was analyzed in an independent experiment as well as CCL20 mean fluorescence intensities **(D)**. Cells were left untreated or stripped with acid buffer to remove non-specifically bound CCL20 from the surface. Flow cytometry plots representative for two experiments with 9 mice in total are displayed **(E)**. *p < 0.05, **p < 0.01.

Since Tfh cells are known to form conjugates with CCR6^+^ B cells, we examined surface expression of CCL20 on these cells. After eliminating autofluorescence (Figure S3 in Supplementary Material) and non-specific binding through isotype and fluorescence-minus-one controls, we show that compared with non-Tfh cells, approximately 4% of Tfh^int^ and Tfh cells show CCL20 on their surface (Figures 2A,B). To eliminate the possibility of the flow cytometric assay detecting bound CCL20 to CCR6, we used CCR6^−/−^ mice and repeated the experiments. CCR6^−/−^ mice similarly demonstrated surface CCL20 expression indicating that CCL20 is bound independent of CCR6 (Figure 2B). The percentage of T cells with surface-bound CCL20 did not differ significantly between parent populations of WT and CCR6^−/−^ mice (Figures 2B,C).

To validate our differential flow cytometric detection of intracellular versus surface CCL20, we analyzed CCL20 expression on *in vitro*-generated Th17 cells (23, 24). As expected, after activation, a major percentage of Th17 cells (circa 90% of Th17 cells) display a strong expression of intracellular CCL20 as published previously (5). However, additionally we were able to detect surface CCL20 on about 25% of Th17 cells (Figure S4 in Supplementary Material).

Chemokines are known to bind sulfated polysaccharides [glycosaminoglycans (GAGs)] found on cell surfaces (25). The presence of GAGs cause polymerization of chemokines, thereby increasing local concentration and facilitating chemokine-receptor binding (26). In this case, the surface-bound CCL20 likely functions to increase local concentration of the chemokine in order to create a concentration gradient for CCR6^+^ cells [haptotaxis (27)]—a likely candidate being partnering CCR6^+^ B cells to T cells as T–B cell conjugates.

The likely source of surface-bound CCL20 appears to be Tfh^int^ and Tfh cells as indicated by protein (Figures 1E,F) and mRNA expression studies (19, 22). However, it is equally possible that CCL20 originates from other sources and becomes tethered on their surfaces. The closely related CCL19 and CCL21, for example, could be detected on the luminal side of high-endothelial venules despite the fact that these cells do not seem to express mRNA for these chemokines. Instead, they rely on transcytosis of the ligands from the surrounding T cell zone, presenting them on their surface to attract CCR7^+^ T cells to secondary lymphoid organs (28, 29). Therefore, it is possible that the source of CCL20 is the GC as both CCR6 and CCL20 are found in GC in lymphoid tissue (30) (Adrian Y. S. Lee and Heinrich Körner, unpublished data). To test whether CCL20 on Tfh^int^ and Tfh cells is covalently expressed on the cell surface or bound *via* GAGs or others, we treated cells with an acid wash before staining. This experiment confirmed the increase in CCL20 expression proportional to Tfh differentiation, with acid wash leaving the frequency of CCL20 expressing cells unchanged (Figure 2C). Unexpectedly, acid wash treatment enhanced the intensity of CCL20 staining (Figure 2D). Careful examination of the histograms showed that acid treatment removed the low-/intermediate surface CCL20 surface expression but not the high-CCL20 expression in the CCL20 positive cell fraction (Figure 2E). Hence, high-CCL20 surface expression represents covalently bound CCL20 and intermediate/low-expression represents CCL20 bound to GAG or others, but likely not in an autocrine or paracrine fashion to CCR6 (see Figure 2B). These data infer that a cell population with a low frequency of CCL20^+^ cells, for instance at a certain differentiation stage such as non-Tfh cells, can eventually provide as many CCL20 ligand binding sites as a population with a higher frequency of CCL20 positive cells. Cells displaying acid-sensitive CCL20 might serve as a mobile source of CCL20, thereby, providing spatio-temporal information during immune responses. Hence, even non-Tfh cells could have the capability of interacting with CCR6-expressing B cells; for instance at the entry point of the GC (12).

### Tfh Cells in T–B Conjugates

**Figure 3:**
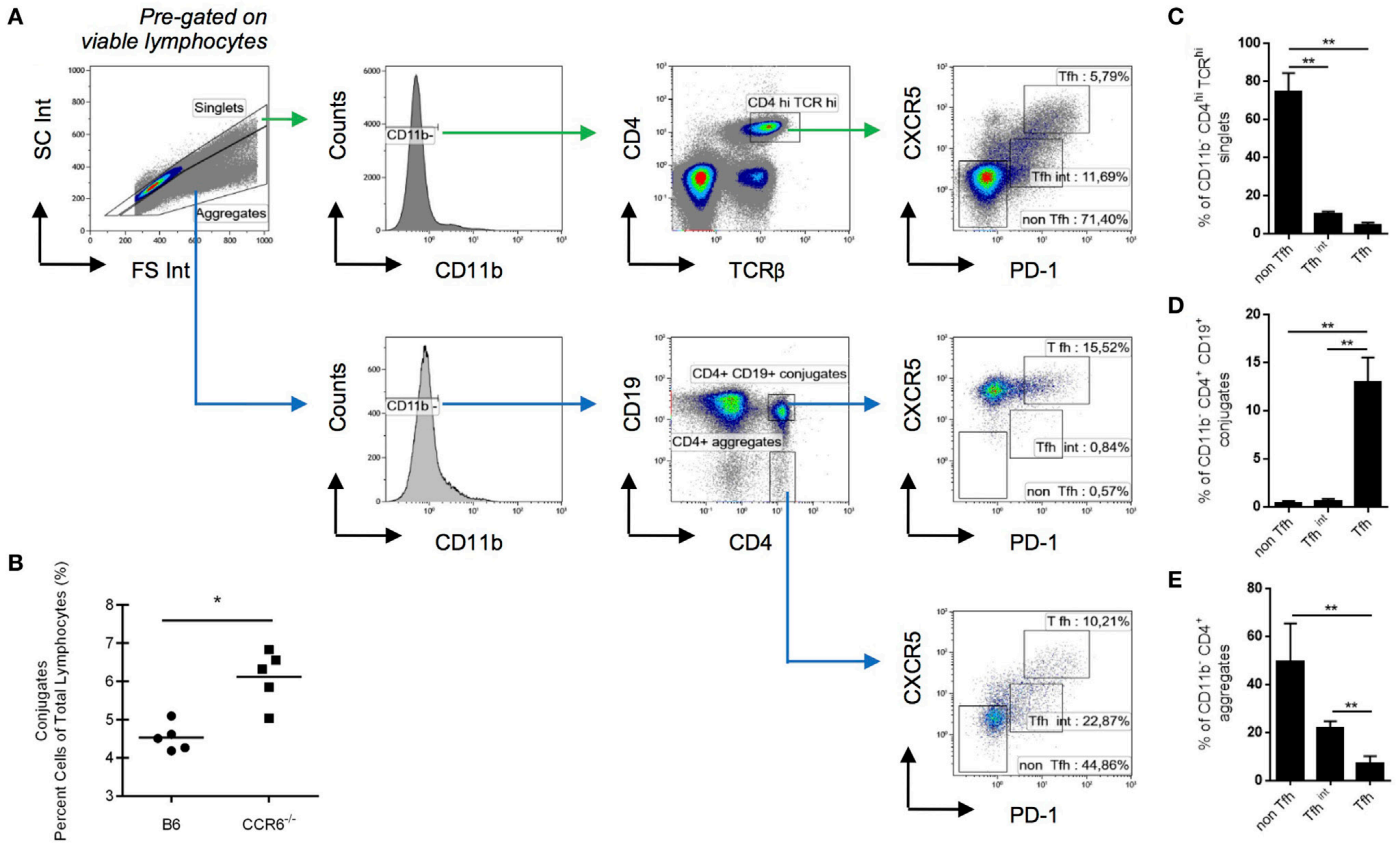
Follicular T helper (Tfh) cells represent the main CD4 subpopulation in T–B cell conjugates. Splenocytes from WT mice were analyzed at day 5 post-sheep red blood cells immunization for the composition of T–B cell conjugates. **(A)** Gating strategy for different CD4 subsets in single cells and in cell aggregates. **(B)** Conjugates are expressed as a percentage of total gated lymphocytes in WT and CCR6^−/−^ mice. **(C)** The frequency of non-Tfh cells (PD-1^−^CXCR5^−^), Tfh^int^ (PD-1^lo^CXCR5^lo^), and Tfh cells (PD-1^hi^CXCR5^hi^) within CD11b^−^ CD4^hi^ TCRαβ^hi^ are shown for singlets. **(D)** Using the same gates for non-Tfh cells, Tfh^in^ and Tfh cells, the frequency of these three subpopulations is shown in CD11b^−^CD4^+^CD19^+^ conjugates and, as a control, in CD11b^−^CD4^+^CD19^−^ conjugates **(E)**, both pre-gated on cell aggregates in the forward-side-scatter. Shown are experiments with five mice {both genotypes; **(B)** or six mice [only wt; **(A,C,D,E)**]}. *p < 0.05, **p < 0.01.

To assess whether this surface-bound CCL20–CCR6 interaction is essential for the formation of T–B cell conjugates, we compared the formation of T–B cell conjugates in SRBC-immunized spleens from WT and CCR6^−/−^ mice by flow cytometry. By gating on doublets and CD4^+^CD19^+^ cells, we determined a surrogate measure of T–B cell conjugates (Figure 3A)—a method performed previously (31). We found a significantly higher proportion of CD4^+^CD19^+^ conjugates in CCR6^−/−^ mice compared with WT mice, indicating that the CCR6–CCL20 chemokine axis is not essential for the formation of these T–B cell conjugates (Figure 3B). This would be in accordance with the observation that mice deficient in CCR6 appeared to form more GCs in an accelerated fashion in the spleen than their WT counterparts (11). To determine which subpopulation of CCL20 expressing T cells (see Figures 2C,D) contributes to T–B-conjugate formation, we analyzed Tfh and Tfh^int^ populations cells in CD11b-negative singlets or aggregates (Figure 3A, gating strategy). Whereas Tfh cells represent only 6–10% of CD4^hi^TCR^hi^ cells within singlets or T cell duplets (Figures 3A,C), they are highly enriched over non-Tfh or Tfh^int^ cells in T–B-conjugates (Figures 3D,E). The stability of the conjugates was addressed in control experiments using acid washing and treatment with EDTA. The proportion of T and B cells in conjugates was not changed by these treatments (data not shown).

Thus, although the presence of CCR6 regulates T–B-conjugate formation negatively (Figure 3B), CCL20^+^ Tfh cells are most enriched in T–B-conjugates. Whether the B cells interacting with CCL20^hi^ Tfh here represent light zone B cells that express CCR6 (17) remains to be tested. In this case, the CCR6–CCL20 axis could be responsible for maintenance of the pairing between Tfh and cognate light zone B cell, thereby, contributing to the selection of high-affinity B cells since CCR6^−/−^ mice display poorer antibody quality production (11, 12).

On the other hand, our data show that Tfh^int^ cells have a relatively high frequency of low-/intermediate CCL20 expressing cells (Figure 2E). It is known that T cells near the T–B border already express Bcl6 and are PD-1^lo^, preceding a Bcl6^+^PD-1^hi^ phenotype (32), and therefore may correspond to our Tfh^int^. This Tfh^int^ population appears to precede cognate B cell interaction (33) and appears to be a necessary step in the selection of B cells and hence, formation of a GC (34). This notion together with our data implicates a fine-tuned CCR6–CCL20 axis in this critical conjugation, which might be modulated by the presentation of non-covalently CCL20 on the surface. Thus, the CCL20–CCR6 axis likely functions at several GC checkpoints governing the efficacy of the GC reaction *via* B cells. It is tempting to speculate that two different mechanisms may act at the GC entry and at the GC selection point, depending on quantity and quality of surface-presented CCL20.

To conclude, we have shown that CCL20 is a chemokine that is produced by monocytes and T cells in the spleen. As subsets of the latter, Tfh and Tfh^int^ cells are producers of CCL20 which is also tethered on their surfaces, an observation we have validated *in vitro* using Th17 cells. A likely function of this CCL20 is in the role of coordinating cognate CCR6^+^ B cells to receive T cell help for the formation of antibodies. As optimal humoral immune responses rely on this critical T–B cell interaction, further research into the role of the CCR6–CCL20 chemokine axis in this conjugation will be helpful in understanding suboptimal immune responses and the pathogenesis of autoimmune disease.

## Ethics Statement

Animal experiments were approved by the University of Tasmania Animal Ethics Committee (A13934—Immunization in C57BL/6 and substrains).

## Author Contributions

AL: designed experiments, carried out experiments, acquired data, analyzed data, wrote the manuscript, and edited the manuscript. DR: designed experiments, carried out experiments, acquired data, analyzed data, and edited the manuscript. AZ and ML: carried out experiments, acquired data, analyzed data, and edited the manuscript. DM: designed experiments, carried out experiments, acquired data, analyzed data, edited the manuscript, and revised the final version. HK: conception of the project, designed experiments, analyzed data, wrote the manuscript, edited the manuscript, and revised the final version.

## Conflict of Interest Statement

The authors declare that the research was conducted in the absence of any commercial or financial relationships that could be construed as a potential conflict of interest.

## Supplementary Material

Figure S1 CC chemokine ligand 20 (CCL20) mRNA becomes upregulated in splenocytes upon immunization and is highly expressed in splenic monocytes and T cells. **(A)** Time course of total spleen *Ccl20* mRNA expression after SRBC intraperitoneal immunization. Graph is representative of six to eight mice at each timepoint. **(B)** *Ccl20* mRNA expression in splenic cellular subpopulations. Day 5 SRBC-immunized spleens were sorted for the four subpopulations. Intact splenocytes were used for “total spleen” and expression was measured relative to *Actb*. Data from three mice and one experiment are shown. NTC, no-template control; ND, not detected. ***p* < 0.01, ****p* < 0.001.

Figure S2 Upregulation of Tfh cells after mice immunization. B6 mice were immunized with intraperitoneal sheep red blood cells and splenocytes subjected to flow cytometry. A clear upregulation of Tfh cells can be detected after 5 days of immunization. Shown here are representative flow plots from five independent experiments.

Figure S3 Method of gating out autofluorescence in flow cytometry. To eliminate the possibility of non-specific fluorescence contributing to apparent cell surface chemokine expression, only cells negative for unutilized fluorescent channels were gated in for analyses (sample plot shown).

Figure S4 CC chemokine ligand 20 (CCL20) and Th17 cells. Assessment of CCL20 expression of Th17 cells in relation to cell proliferation was performed using flow cytometry. Isolated CD4^+^ lymph node T cells were labeled with cell trace violet (CTV) and were activated *in vitro* with CD3/CD28 in the presence of a cocktail of TGF-β, IL-6, IL-23 in combination with anti-IL-4 and anti-IFN-γ for 72 h following standard protocols. The expression of CCL20 on the surface **(A)** and intracellularly **(B)** was detected using a directly labeled anti-CCL20 mAb. The expression of IL-17 was independently verified using intracellular flow cytometry and is not shown. A representative result is shown.
