# Supplementary figures and images for: Expression of Membrane-Bound CC Chemokine Ligand 20 on Follicular T Helper Cells in T–B-Cell Conjugates
Source: Front Immunol. 2017 Dec 21;8:1871. doi: 10.3389/fimmu.2017.01871 (PMC5763129; doi:10.3389/fimmu.2017.01871)

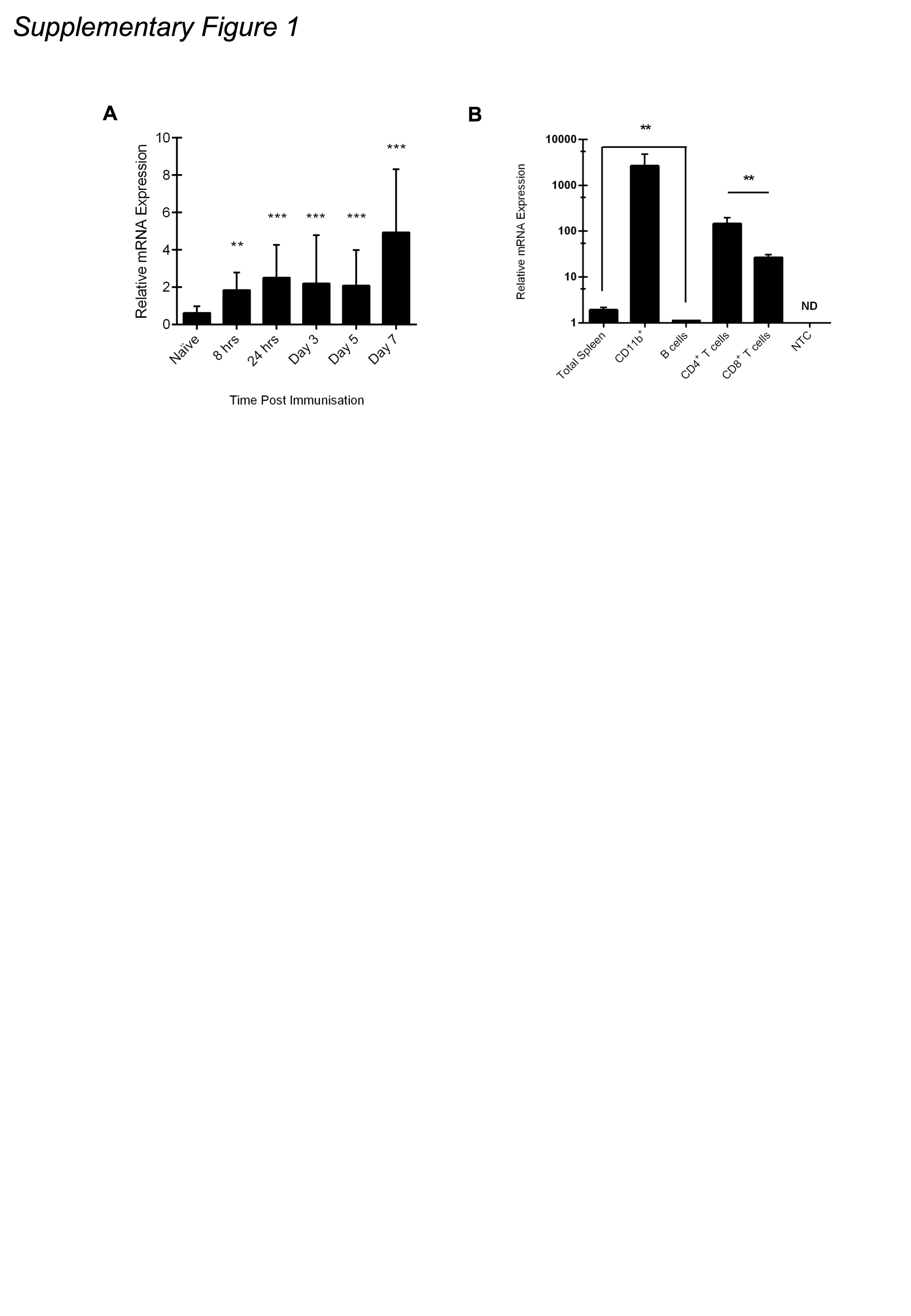

Supplement: Figure S1 — CC chemokine ligand 20 (CCL20) mRNA becomes upregulated in splenocytes upon immunization and is highly expressed in splenic monocytes and T cells. (A) Time course of total spleen Ccl20 mRNA expression after SRBC intraperitoneal immunization. Graph is representative of six to eight mice at each timepoint. (B) Ccl20 mRNA expression in splenic cellular subpopulations. Day 5 SRBC-immunized spleens were sorted for the four subpopulations. Intact splenocytes were used for “total spleen” and expression was measured relative to Actb. Data from three mice and one experiment are shown. NTC, no-template control; ND, not detected. **p < 0.01, ***p < 0.001. [file Image_1.jpeg]

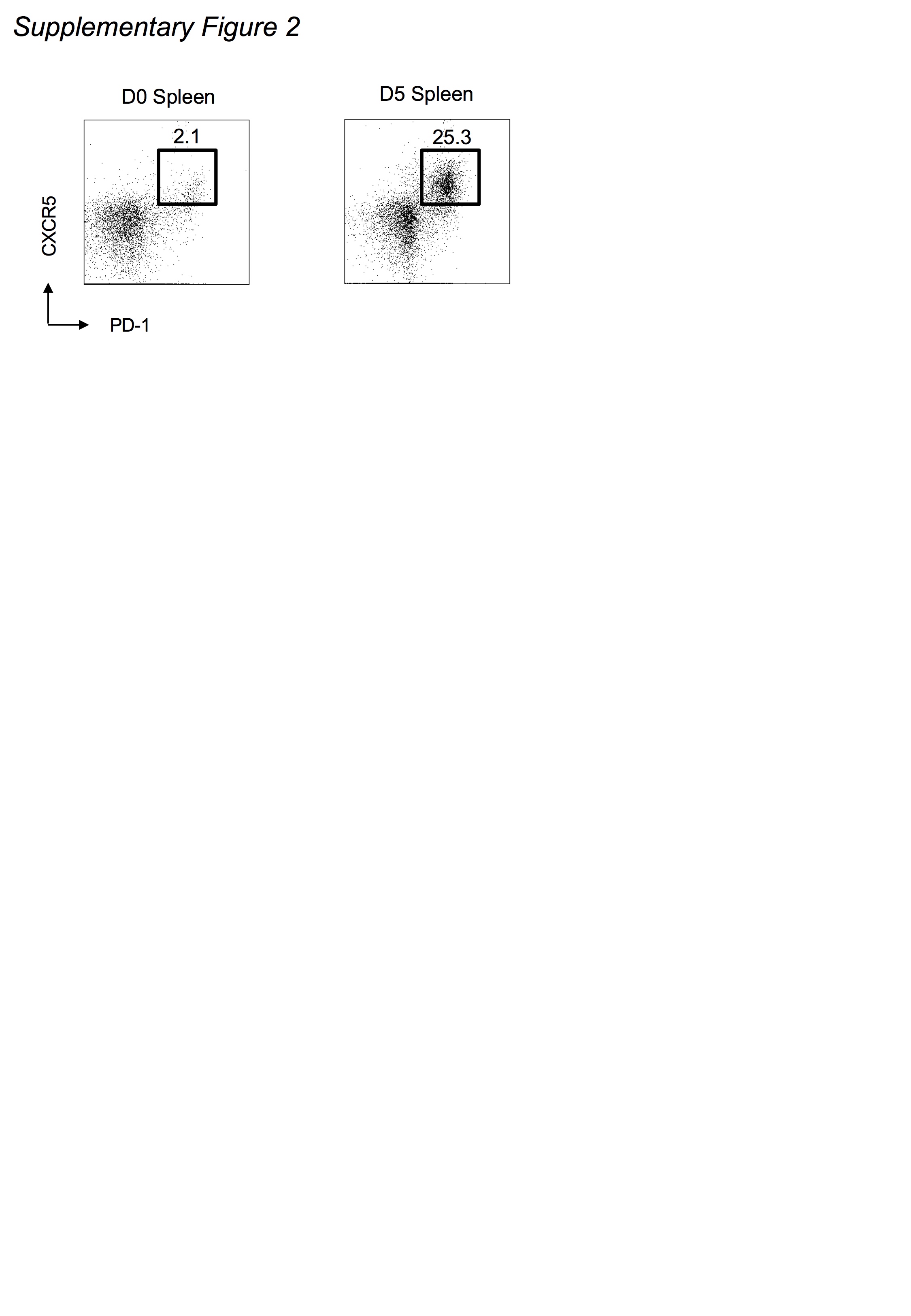

Supplement: Figure S2 — Upregulation of Tfh cells after mice immunization. B6 mice were immunized with intraperitoneal sheep red blood cells and splenocytes subjected to flow cytometry. A clear upregulation of Tfh cells can be detected after 5 days of immunization. Shown here are representative flow plots from five independent experiments. [file Image_2.jpeg]

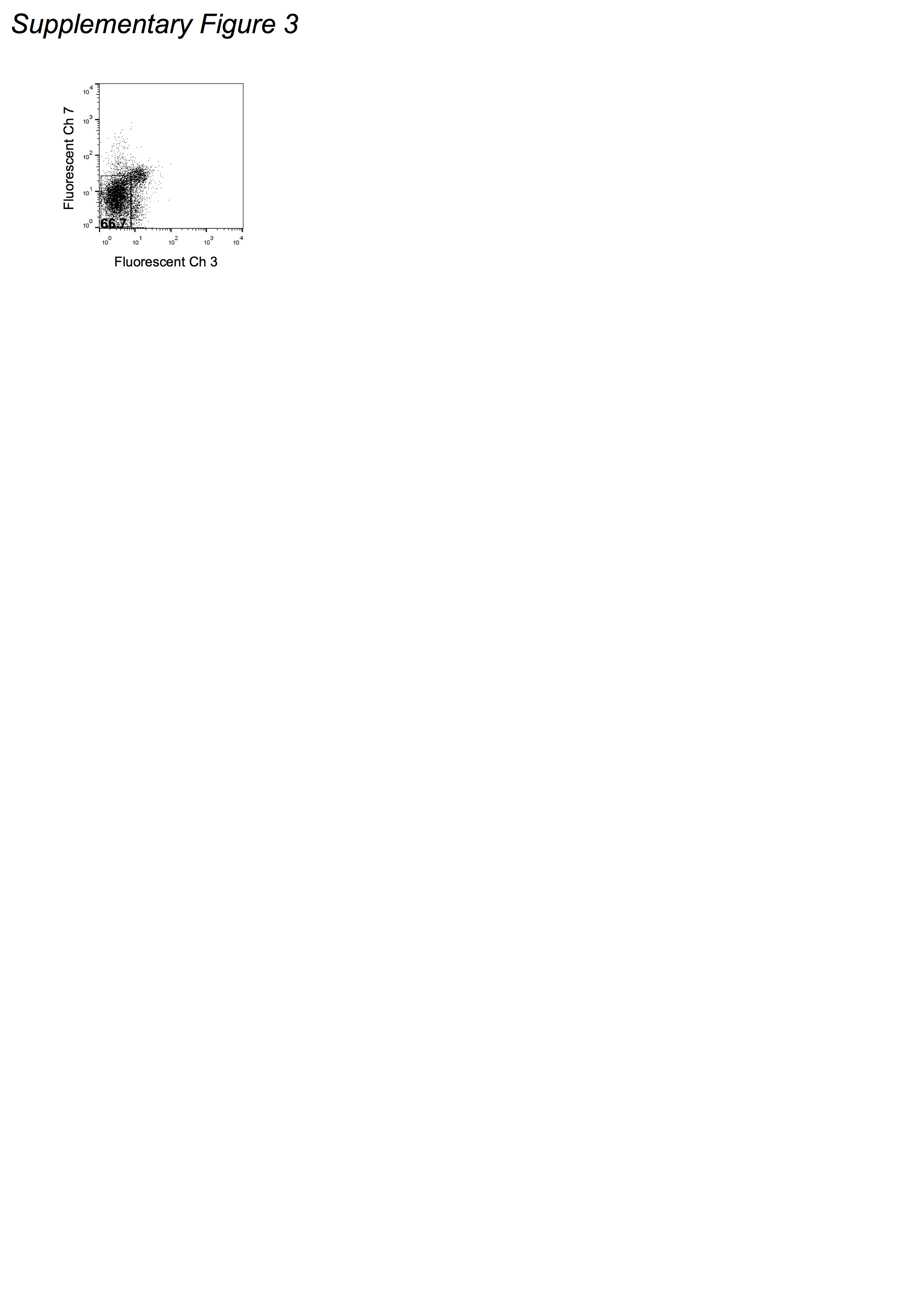

Supplement: Figure S3 — Method of gating out autofluorescence in flow cytometry. To eliminate the possibility of non-specific fluorescence contributing to apparent cell surface chemokine expression, only cells negative for unutilized fluorescent channels were gated in for analyses (sample plot shown). [file Image_3.jpeg]

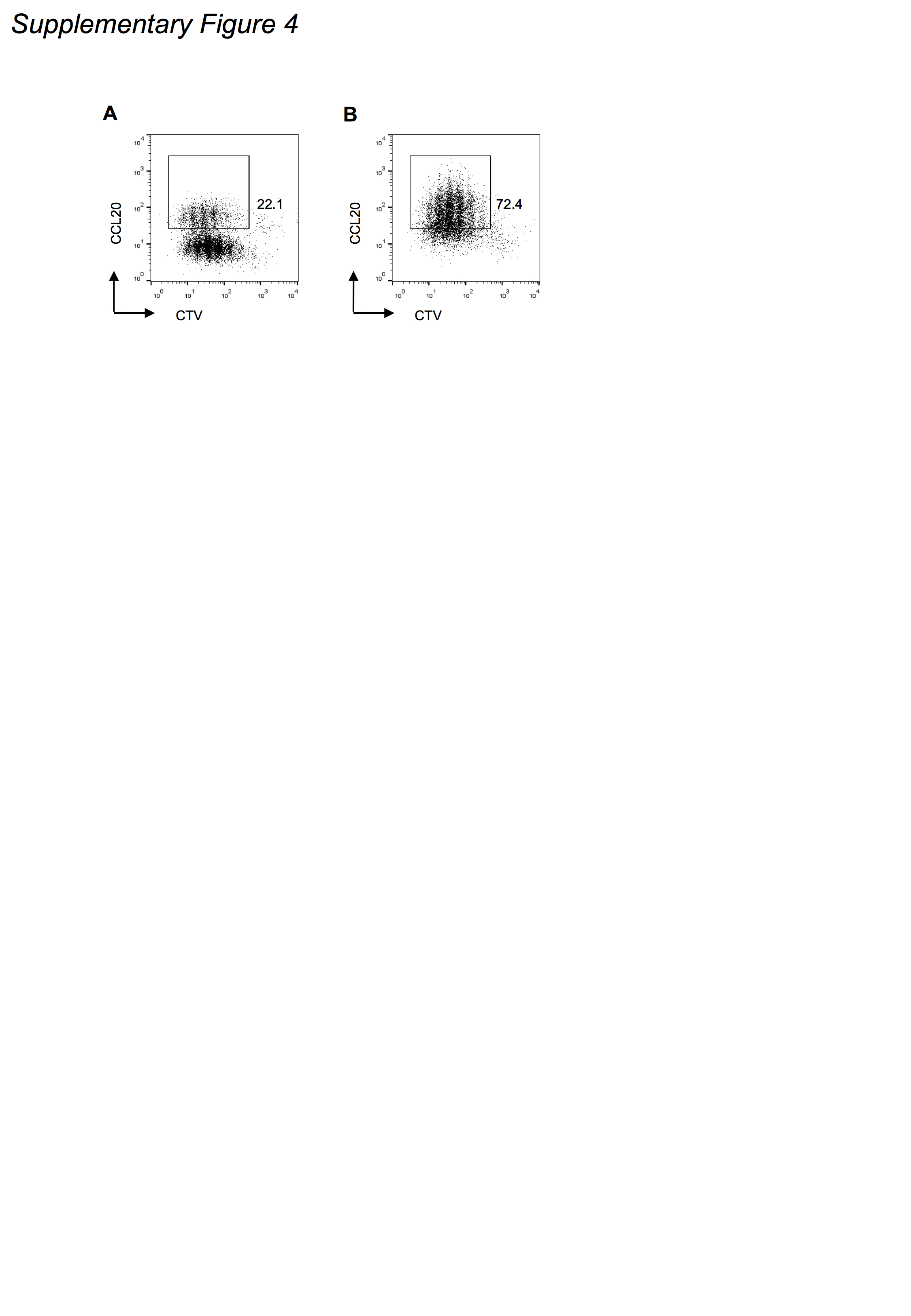

Supplement: Figure S4 — CC chemokine ligand 20 (CCL20) and Th17 cells. Assessment of CCL20 expression of Th17 cells in relation to cell proliferation was performed using flow cytometry. Isolated CD4+ lymph node T cells were labeled with cell trace violet (CTV) and were activated in vitro with CD3/CD28 in the presence of a cocktail of TGF-β, IL-6, IL-23 in combination with anti-IL-4 and anti-IFN-γ for 72 h following standard protocols. The expression of CCL20 on the surface (A) and intracellularly (B) was detected using a directly labeled anti-CCL20 mAb. The expression of IL-17 was independently verified using intracellular flow cytometry and is not shown. A representative result is shown. [file Image_4.jpeg]
